# PREVALENCE, BURDEN, AND LOCATION OF CEREBRAL MICROINFARCTS AND THEIR ASSOCIATION WITH LATE-LIFE BLOOD PRESSURE

**DOI:** 10.1093/geroni/igac059.2745

**Published:** 2022-12-20

**Authors:** Mo-Kyung Sin, Yan Cheng, Ali Ahmed, Jeffrey Roseman, Edward Zamrini

**Affiliations:** Seattle University, Seattle, Washington, United States; George Washington University, Washington, District of Columbia, United States; Washington DC Veterans Medical Center, Washington DC, District of Columbia, United States; University of Alabama at Birmingham, Birmingham, Alabama, United States; Irvine Clinical Research, Irvine, California, United States

## Abstract

Little is known about the relationship of late-life blood pressure (BP) profiles (level, trend and variability) with the presence, number and regional distribution (cortical vs subcortical) of cerebral microinfarcts. We examined these associations in older adults (age ≥65) using the Adult Changes in Thought (ACT) data. 551 participants (94.6% ≥80 years, 58.6% women, 94.2% white) had complete data on microinfarcts and 4 BP measures. Late-life BP, defined by the four BP values before death (2 year-time gap between measures), was treated using 3 different approaches: (1) categories (based on mean of 4 values); (2) trend (based on the difference between values 1 and 4); and (3) variability (based on standard deviation of the mean of the changes in 4 BP values from 1 to 4). Multivariable-adjusted logistic regression models were used to examine the associations of the late-life BP with microinfarcts, adjusting for relevant covariates. Each of the 5 systolic BP categories between 120 and 169 mmHg (reference: 110–119 mmHg) had significant twice higher odds (4 times for SBP ≥170) of presence and number of microinfarcts, which was only significant for subcortical region. Similar higher odds were observed for systolic BP trend (increase/decrease/no change), which was significant for both cortical and subcortical microinfarcts. Systolic BP variability had no significant association with microinfarcts. Similar associations were observed with diastolic BP. In conclusion, the presence (especially for subcortical regions) and number of microinfarcts were more likely to be in those with higher mean BP and those whose BP changed over the study.

